# A Secure and Efficient Group Key Management Protocol with Cooperative Sensor Association in WBANs

**DOI:** 10.3390/s18113930

**Published:** 2018-11-14

**Authors:** Haowen Tan, Ilyong Chung

**Affiliations:** Department of Computer Engineering, Chosun University, Gwangju 61452, Korea; tan_halloween@foxmail.com

**Keywords:** wireless body area networks, group key management, authentication, Chinese remainder theorem (CRT), coded cooperative data exchange (CCDE)

## Abstract

The wireless body area network (WBAN) is considered as one of the emerging wireless techniques in the healthcare system. Typical WBAN sensors, especially implantable sensors, have limited power capability, which restricts their wide applications in the medical environment. In addition, it is necessary for the healthcare center (HC) to broadcast significant notifications to different patient groups. Considering the above issues, in this paper, the novel practical WBAN system model with group message broadcasting is built. Subsequently, a secure and efficient group key management protocol with cooperative sensor association is proposed. In the proposed protocol, the Chinese remainder theorem (CRT) is employed for group key management between HC and the personal controller (PC), which also supports batch key updating. The proposed sensor association scheme is motivated by coded cooperative data exchange (CCDE). The formal security proofs are presented, indicating that the proposed protocol can achieve the desired security properties. Moreover, performance analysis demonstrates that the proposed protocol is efficient compared with state-of-the-art group key management protocols.

## 1. Introduction

Development of wireless communication and sensor technologies has enabled remarkable improvement in both academic research and practical applications of wireless body area networks (WBAN), which offer ubiquitous wireless communication services to users [1]. In the medical field, WBAN is used to monitor patients’ real-time health status and seamlessly transmit physiological data to medical institutions including hospitals, community clinics and emergency centers. Consequently, the doctor could conduct remote diagnostics on the patients and provide timely medical assistance. Additionally, with necessary symptom detection, early warnings, as well as precautionary measurements for certain diseases including asthma, AIDS, cancer and influenza can be provided [2].

Nowadays, as a crucial part of the Internet of Thing (IoT), WBANs have continuously attracted much attention. Its architecture varies greatly, so as to adjust to diverse requirements of different practical scenarios. In general, a typical WBAN designed for the healthcare system mainly consists of the healthcare center (HC), personal controller (PC) and many low-power wireless medical sensors implanted inside or attached to the patient’s body [1]. Through these sensors, vital biomedical information such as heartbeat and blood pressure can be measured and then transmitted to the healthcare center (HC) through the personal controller (PC). Therefore, the doctor or physician could be aware of a patient’s real-time physical parameters by analyzing the acquired biomedical information. According to these analysis results, appropriate remote diagnostics and timely medical assistance are provided. Note that HC here refers to the healthcare service provider such as a hospital or clinic. PC is a mobile device responsible for both biomedical information gathering from sensors and communication with HC. It is assumed that each patient is combined with one PC. Particularly, in numerous scenarios, the role of PC is normally played by PDAs or smartphones. The applications and implementations of WBANs offer a better choice of receiving healthcare services for patients or other people who need to be taken good care of, for example aged or disabled people in need of long-term physiological monitoring.

The sensitive biomedical data transmission is through an open wireless channel, where the patients’ private physical condition may be revealed to an unauthorized entity. Consequently, appropriate strategies are urgently required to guarantee enough security properties and privacy protections.

In practical WBAN scenarios, the HC is responsible for providing medical services to large numbers of patients simultaneously [3,4]. Meanwhile, the patients with different diseases are allocated to different departments. For instance, patients with coronary disease are arranged with the cardiovascular department, while patients with skin disease such as allergic dermatitis are arranged with the dermatological department. In consideration of this, it is essential for the HC to provide push notification service to patients of different departments, respectively. Patients of the same department could also exchange their information on certain diseases. Therefore, a specific group communication channel between HC and patients is indispensable, which enables notification broadcast and information exchange between HC and patients. Moreover, the patients may frequently join or leave the healthcare system. In this way, efficient group key management employing join and revocation operation is of great significance [5]. With the generated group key, HC could broadcast notifications to a specific patient group, and patients of this group could communicate with each other as well [6].

WBAN sensors, including implantable sensors and wearable sensors, are low-power wireless devices with limitations on computation, communication, power and storage [1,2]. In particular, for implantable sensors, which are designed to be implanted in the human body in order to monitor essential physiological parameters, it is impractical to frequently recharge or change the built-in battery. Meanwhile, an increased computation and transmission load on the sensor side can dissipate more power into heat and eventually do harm to human organs [7]. In this assumption, the transmission passes, as well as the computation load, should be considerably optimized for the purpose of prolonging the working time period and preventing the human body from the thermal effect [8]. As a result, an efficient group key generation and management for implantable sensors is of great significance [3,9].

In this paper, we propose an efficient cooperative sensor association and group key management protocol with the Chinese remainder theorem (CRT) in wireless body area networks. Our nontrivial efforts can be summarized as follows:

1. A novel WBAN model with message broadcasting: In practical medical WBAN scenarios, patients who receive services from HC are allocated to different departments according to their physical conditions and diseases. As a result, it is necessary for HC to provide a notification service to different patient groups. To the best of our knowledge, we are the first to propose the system model providing a specific group communication channel for message broadcasting between HC and patients. Moreover, the medical data transmission channel from sensors to PC is also taken into consideration in our design.
2. Group key management between HC and PC with CRT: The Chinese remainder theorem is employed for the group key management between HC and PC, which also supports batch key updating. In this case, HC is capable of broadcasting messages to different patient groups. Moreover, patients in the same group are capable of exchanging information about their physical conditions.
3. Group key management between PC and sensors with CCDE: In our design, the group key management between PC and sensors is motivated by coded cooperative data exchange for the purpose of minimizing the communication rounds for group key generation. Hence, the communication and computation complexity can be drastically reduced, which is efficient for resource-limited wireless sensors in WBAN.

The remainder of this paper is organized as follows. Section 2 briefly surveys the relevant research achievements. Section 3 introduces some necessary preliminary works and the designed system model in order to allow the reader to obtain a better understanding of the topic. Section 4 presents the proposed sensor association and group key management protocol in detail. Section 5 demonstrates the security analysis. Section 6 displays the performance analysis. The conclusion is drawn in Section 7.

## 2. Related Works

To the best of our knowledge, many research achievements have been made on group key management for wireless body area networks. Theoretically, the traditional public key cryptosystem (TPKC) had been implemented in wireless body area networks previously [10,11,12,13,14,15]. A certificate generated by a third party is required to combine the identity of the user and the associated public key. However, in TPKC-based schemes, complex modular exponentiation is calculated so that more computation and storage are required in resource-limited wireless sensor devices. Therefore, these TPKC-based group key management schemes cannot meet the practical requirements. In order to alleviate the computation and storage burden on the sensor side, several authentication and group management schemes [4,16,17,18] based on elliptic curve cryptography (ECC) have been proposed, which provide the same security with a smaller key size compared to TPKC-based schemes.

Many researchers applied the idea of identity-based public key cryptography (ID-PKC) [19], which was a cryptography technique first proposed by Shamir [20] in order to address the certificate management problem in TPKC. In ID-PKC, the public key of the user can be calculated from his/her publicly-known identity, while the secret key of each user is generated by a fully-trusted key generation center (KGC). In 2009, Yang et al. [21] proposed an ID-PKC-based key management scheme for mobile devices. However, Yoon and Chang [22] proved that the proposed scheme was vulnerable to impersonation attacks. Subsequently, several ID-based key agreement protocols were proposed [23,24,25].

Certificateless public key cryptography (CL-PKC) was first introduced by Al-Riyami and Paterson [26] in 2003. In CL-PKC, the private key of the user consists of two parts, which are respectively generated by a semi-trusted key generation center (KGC) and by the user himself/herself. Hence, the key escrow problem, as well as the certificate management problem can be addressed. Liu et al. [2] proposed two certificateless authentication protocol in the WBAN environment. However, Xiong [27] demonstrated that Liu et al.’s protocols could not provide forward security and scalability. Additionally, a new certificateless encryption scheme and the signature scheme with efficient revocation against short-term key exposure were proposed in [28]. Thereafter, He et al. [3] proposed an efficient certificateless public auditing (CLPA) scheme with the purpose of addressing integrity issues in cloud-assisted WBANs.

Furthermore, the Chinese remainder theorem (CRT) has been applied in many existing group key distribution schemes [29,30,31,32]. Zheng et al. proposed two centralized group key management protocols based on CRT [29]. The main contribution of this work is that the transmission passes for group key distribution are minimized, which is available in wireless networks with sourced restriction. After that, Zhou et al. proposed a key tree and CRT-based group key distribution scheme [30]. Note that in this scheme, the key server uses the root keys of the group member subtrees and CRT for group key distribution. Moreover, the computation on the user side is minimized. Based on this, Vijayakumar et al. proposed CRT-based centralized group key management for secure multicast communication [33]. The proposed key management scheme could prominently reduce the computation complexity of the key server.

Coded cooperative data exchange (CCDE) as first introduced by Rouayeb et al. [34] in 2010 and has drawn increasing attention [35,36,37]. Milosavljevic et al. proposed a deterministic algorithm for CCDE [38], where a novel divide and conquer-based architecture was presented in order to determine the number of bits each node should transmit in the public channel. Subsequently, Sprinston et al. [39] presented a randomized algorithm with a high probability to minimize the number of transmissions over the public channel. In 2016, Courtade et al. characterized the minimum number of public transmissions for key agreement [40] with an arbitrary key distribution.

The aforementioned group key management schemes vary greatly with different security techniques. The existing research emphasizes the secure data transmission between sensors and PC, while the communication and access control for patients remain to be enhanced. In this paper, we design an integral system model involving both HC-PC and PC-sensor communication. In practical scenarios, a high turnover of patients brings frequent key updating in the hospital environment. In this case, we adopt the CRT to PC group key distribution, which could provide fast and effective key updating. Additionally, the CCDE is adopted in sensor group key distribution. Note that the decentralized cooperative key generation strategy drastically decreases the communication cost, which is suitable for resource-limited WBAN sensors. The corresponding security and performance analysis demonstrates that the proposed protocol could provide adequate security assurance and efficiency.

## 3. Preliminaries and Model Definitions

This section introduces some necessary preliminaries to facilitate the reader’s understanding, including bilinear pairing, the coded cooperative data exchange problem (CCDE) and the Chinese remainder theorem (CRT). Meanwhile, the system model and network assumption are presented.

### 3.1. Bilinear Pairing

Let $G_{1}$, $G_{2}$ and $G_{S}$ be multiplicative cyclic groups of a large prime order P. A map function $\hat{e}:G_{1}\times G_{2}\to G_{S}$ is a bilinear pairing if it satisfies the three properties below:

1. Bilinearity: For $\forall g_{1}\in G_{1}$, $\forall g_{2}\in G_{2}$ and ∀a,b∈Z, there is $\hat{e}\left({g_{1}}^{a},{g_{2}}^{b}\right)=\hat{e}{\left(g_{1},g_{2}\right)}^{ab}$.
2. Non-degeneracy: For $\exists g_{1}\in G_{1}$ and $\exists g_{2}\in G_{2}$, there is $\hat{e}\left(g_{1},g_{2}\right)\neq 1$.
3. Computability: For $\forall g_{1}\in G_{1}$ and $\forall g_{2}\in G_{2}$, there exists an efficient algorithm to compute $\hat{e}\left(g_{1},g_{2}\right)$.

### 3.2. Coded Cooperative Data Exchange Problem

A set $X=\{x_{1},∆,x_{n}\}$ of *n* packets each belonging to a finite alphabet A needs to be delivered to a set of *k* clients $C=\{c_{1},∆,c_{k}\}$. Each client $c_{i}\in C$ initially holds a subset $X_{i}$ of packets denoted by $X_{i}\subseteq X$. We denote by $n_{i}=\left|X_{i}\right|$ the number of packets initially available to client $c_{i}$ and by $\bar{X_{i}}=X\backslash X_{i}$ the set of packets required by $c_{i}$. We assume that the clients collectively know all packets in *X* ($\cup_{c_{i}\in C}X_{i}=X$). Each client can communicate to all its peers through an error-free broadcast channel capable of transmitting a single packet in A. The data are transmitted in communication rounds. For example, in round *i*, one of the clients $c_{j}$ broadcasts a packet *x* to all its outgoing neighbors in *C*. The transmitted information *x* may be one of the original packets in $X_{j}$ or some encoding of packets in $X_{j}$ and the information previously transmitted to $c_{j}$ [34,37].

The problem is to find a scheme that enables each client $c_{i}\in C$ to obtain all packets in $\bar{X_{i}}$ (and thus, in *X*) while minimizing the total number of broadcasts [35].

### 3.3. Chinese Remainder Theorem

Let $k_{1}$, ..., $k_{n}$ be positive integers that are relatively prime in pairs. Then, for any given integers $a_{1}$, ..., $a_{n}$, the system of congruences:

$$X\equiv {\left\{a_{i}\;\mathrm{mod}\;k_{i}\right\}}_{i\in [1,n]}$$

has a unique solution modulo $\partial_{g}=\prod_{i=1}^{n}k_{i}$. The solution is given by:

$$C\equiv \sum_{i=1}^{n}\alpha_{i}\beta_{i}\gamma_{i}\;\mathrm{mod}\;k_{i},$$

where $\beta_{i}=\frac{\partial_{g}}{k_{i}}$ and $\beta_{i}\times \gamma_{i}\equiv 1\;\mathrm{mod}\;k_{i}$.

### 3.4. System Model

**Figure 1:**
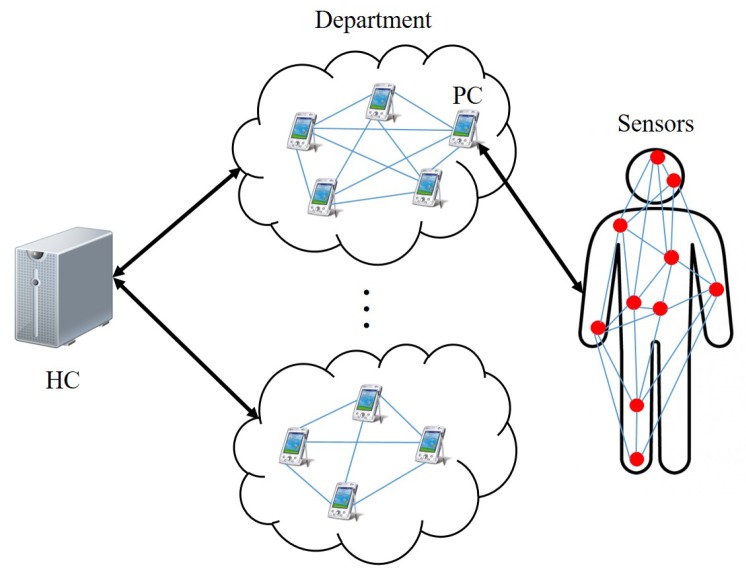
System model.

As shown in Figure 1, the entire system model consists of three entities: the healthcare center (HC), the personal controller (PC) and the sensors. The description of these three entities is given below.

The healthcare center (HC) is a trustworthy authority providing medical service to the patients. HC is assumed to have adequate storage and computation power. In our system model, HC communicates with PCs to obtain physiology data of patients. Hence, the patient’s physical condition can be remotely monitored.

The personal controller (PC) is a mobile device responsible for both biomedical information gathering from sensors and communication with HC. Note that each patient is combined with one PC. The PC employed in this paper is assumed to be professional equipment designed specifically for medical purposes.

Sensors are low-power wireless medical devices either implanted inside or attached to a patient’s body. These sensors have limited computation ability and restricted battery capacity. The sensors are responsible for real-time measurement of various physiological parameters of patients.

### 3.5. Network Assumption

According to Figure 1, there are several departments in the healthcare center. Patients with different diseases are assigned to different departments. In each department, the patients are arranged to be one patient group. HC is assumed to provide service to all the departments (patient groups).

A secure communication channel for data transmission between HC and PC is essential. Furthermore, as mentioned above, a specific group communication channel between HC and a particular patient group is indispensable. As for individual patient, the secure association between PC and multiple sensors is crucial so that the vital physical data from sensors can be safely transmitted.

In our system model, the PC is designed to communicate directly with HC through a wireless channel, which is different from other existing WBAN models using Internet communication between PC and HC [3,28]. PC is designed as a professional medical device with appropriate treatment units. As part of the medical facility, it is assumed that PC works within the effective range of HC [41,42]. After the patient fully recovers from the disease, his/her PC will be removed and arranged with other new patients.

## 4. Proposed Schemes

In this section, we explain our cooperative sensor association and group key management protocol, which can be generally divided into two parts: the group key management between HC and PCs affiliated with the same patient group and the cooperative association between sensors and the related PC. According to Figure 1, we assume that HC is in charge of *r* departments in total. Each department consists of multiple PCs. In this case, one PC is combined with one patient. Consequently, the patient and the relevant PC in this paper are considered as one entity. In department *j* (j∈[1,r]) with *n* PCs (patients) in total, ${\mathrm{PC}}_{i}$ (i∈[1,n]) is in contact with the corresponding patient $P_{i}$. As for $P_{i}$, *m* sensors are arranged in or on different parts of $P_{i}$’ body in order to monitor various physiological parameters.

In our design, we are motivated to build a group key management scheme between HC and all the *n* PCs in department *j*. At the same time, group key agreement between ${\mathrm{PC}}_{i}$ and the *m* sensors is provided accordingly. We introduce our protocol based on department *j*. Meanwhile, the design for the multiple department situation is similar. The notations used in our protocol are described in the following subsection. Thereafter, the detailed description of our protocol is given, which contains four parts: group key generation for HC and PCs, PC join and leave operation, group key generation for PC and sensors and sensor join and leave operation.

### 4.1. Notations

The notations used in our protocol and a brief description are listed in Table 1.

### 4.2. Group Key Generation for HC and PCs

In this section, the group key generation for HC and PCs affiliated with department *j* is described. It is worth noting that the generation procedures for multiple departments are similar. The proposed group key generation for HC and PCs can be divided into three phases. The first phase is the registration phase, which is responsible for secret key allocation to each PC and other necessary precomputation. The second phase is the group key computation phase, where the group key is generated and distributed to PCs. At last, in the group key derivation phase, each PC derives the group key from the received keying message. The detailed descriptions of these three phases are as follows.

#### 4.2.1. Registration Phase

Before the group key generation procedure, some essential operations should be previously conducted by HC in the registration phase [43]. Initially, let $P_{1}$, ..., $P_{n}$ be *n* patients who are assigned to department *j* (j∈[1,r]). First, patient $P_{i\in [1,n]}$ registers to HC so that HC could acquire $P_{i}$’s personal information including name, age, gender, phone number, and so on. Thereafter, HC allocates ${\mathrm{PC}}_{i}$ to $P_{i}$. Next, HC generates the symmetric key hsk and the secret key $P\;S\;K_{i}$ for ${\mathrm{PC}}_{i\in [1,n]}$ by conducting **SecKeGen**. Subsequently, HC executes **PreCom** for necessary precomputation. The design of **SecKeGen** and **PreCom** is presented below.

●  **SecKeGen**: The HC conducts **SecKeGen** to generate information for ${\mathrm{PC}}_{i\in [1,n]}$. $Z_{p}^{*}$ and $Z_{s}^{*}$ are defined as two nonnegative integers sets less than *p* and *s*, respectively, where *p* and *s* are two large prime numbers. Additionally, G is defined as a multiplicative group of *p*, and *g* is a generator of G. HC randomly chooses SSK and $P\;S\;K_{i\in [1,n]}$ from $Z_{p}^{*}$, where $P\;S\;K_{i}$ is the secret key of ${\mathrm{PC}}_{i}$ and SSK is the HC master key. Moreover, HC chooses $hsk\in Z_{s}^{*}$ for symmetric encryption. As a result, the HC temporary identity HID is generated as:

(1) $$H\;I\;D=g^{S\;S\;K||T\;S},$$

where TS is the current time stamp.

During the registration phase, HC assigns $\langle P\;S\;K_{i},H\;I\;D,hsk\rangle$ to ${\mathrm{PC}}_{i\in [1,n]}$ of department *j* and keeps the master key SSK only in its memory. In other words, HC maintains a key list for each department, where SSK, hsk, HID and $P\;S\;K_{i}$ of *n* PCs are stored. Each ${\mathrm{PC}}_{i}$ possesses $\langle P\;S\;K_{i},H\;I\;D,hsk\rangle$. Note that SSK is the confidential information only known to HC, while HID and hsk are assumed to be known to all PCs in department *j*.

●  **PreCom**: The HC conducts **PreCom** to compute the essential intermediate values [44]. First, HC selects $P\;S\;K_{i}$ from the key list and computes:

(2) $$\partial_{g}=P\;S\;K_{1}\times \ldots \times P\;S\;K_{n}=\prod_{i=1}^{n}P\;S\;K_{i}$$

involving *n* registered PCs of department *j*. Then, for each ${\mathrm{PC}}_{i\in [1,n]}$, HC computes:

(3) $$x_{i}=P\;S\;K_{1}\times \ldots P\;S\;K_{i-1}\times P\;S\;K_{i+1}\times \ldots P\;S\;K_{n}=\frac{\partial_{g}}{P\;S\;K_{i}}$$

and obtains $\left\{x_{1},\ldots ,x_{n}\right\}$. That is, $x_{i}$ for ${\mathrm{PC}}_{i\in [1,n]}$ is the multiplication of all the remaining $P\;S\;K_{i}$. Subsequently, HC computes $y_{i}$ for each $x_{i}$ (i∈[1,n]), which satisfies:

(4) $$x_{i}\times y_{i}\;\mathrm{mod}\;P\;S\;K_{i}\equiv 1.$$

That is, $y_{i}$ is the modular multiplicative inverse of $x_{i}$ to the modulus $P\;S\;K_{i}$. Hereafter, HC acquires the variables $var_{i\in [1,n]}$ according to:

(5) $$var_{i}=x_{i}\times y_{i}.$$

Thus, the intermediate value μ can be computed as:

(6) $$\mu =var_{1}\times \ldots \times var_{n}=\sum_{i=1}^{n}var_{i}.$$

Upon completion, HC stores the value of μ for the following group key computation. At this point, the precomputation based on CRT is completed.

#### 4.2.2. Group Key Computation Phase

In this phase, the group key of department *j* is generated by HC. Let *q* be a large prime number where $q\leq \left\lceil p/2\right\rceil$. First, HC chooses a random value from $Z_{p}^{*}$ as the group key $P\;G\;K_{j}$. Then, **PGKCom** is conducted by HC in order to obtain the keying message. Finally, HC conducts **SecHtoP** to distribute the keying message to all ${\mathrm{PC}}_{i\in [1,n]}$. The design of **PGKCom** and **SecHtoP** is described in detail below.

●  **PGKCom**: In our design, the HC conducts **PGKCom** to get the keying message $\gamma_{j}$ for department *j*, which is illustrated as:

(7) $$\gamma_{j}=P\;G\;K_{j}\times \mu .$$

Particularly, for department *j*, only one $P\;G\;K_{j}$ and one μ are effective in the same time interval. Furthermore, the keying message $\gamma_{j}$ is available for all PCs.

●  **SecHtoP**: The HC conducts **SecHtoP** to distribute the keying message $\gamma_{j}$ to department *j*. First, HC encrypts the keying message, illustrated as:

(8) $$E\left(\gamma_{j}\right)=S\;\_E\;N\;C_{hsk}\left(T\;S|\right|\gamma_{j}),$$

where $S\;\_E\;N\;C_{x}\left(M\right)$ denotes the symmetric encryption using *x*. Next, HC computes the certificate $S\;I\;G_{S\;S\;K}(T\;S||H\;I\;D||E\left(\gamma_{j}\right))$ according to:

(9) $$S\;I\;G_{x}\left(T\;S||M\right)=H{\left(M\right)}^{x||T\;S}.$$

In this way, the certificate can be obtained as:

(10) $$S\;I\;G_{S\;S\;K}(T\;S||H\;I\;D||E\left(\gamma_{j}\right))=H(H\;I\;D||E\left(\gamma_{j}\right){)}^{S\;S\;K||T\;S}.$$

Following the above calculation, the message:

$$\langle T\;S||H\;I\;D||E\left(\gamma_{j}\right)||S\;I\;G_{S\;S\;K}(T\;S||H\;I\;D||E\left(\gamma_{j}\right))\rangle$$

is finally broadcast to ${\mathrm{PC}}_{i\in [1,n]}$ of department *j*.

#### 4.2.3. Group Key Derivation Phase

In this phase, the main task for ${\mathrm{PC}}_{i}$ is to verify the validity of the received message by employing **AuthMess**. Subsequently, ${\mathrm{PC}}_{i}$ derives the group key $P\;G\;K_{j}$ using **GrKeCom**. The design of **AuthMess** and **GrKeCom** is described in detail below.

●  **AuthMess**: ${\mathrm{PC}}_{i}$ conducts **AuthMess** to verify the received message from HC. First, ${\mathrm{PC}}_{i}$ checks the time stamp TS from the broadcast message. If TS matches the current time, ${\mathrm{PC}}_{i}$ checks whether:

$$\hat{e}(S\;I\;G_{S\;S\;K}(T\;S||H\;I\;D||E\left(\gamma_{j}\right)),g)\overset{?}{=}\hat{e}(H(H\;I\;D||E\left(\gamma_{j}\right)),H\;I\;D)$$

holds. The correctness is elaborated as follows:

(11) $$\begin{array}{c} \;\;\;\hat{e}(S\;I\;G_{S\;S\;K}(T\;S||H\;I\;D||E\left(\gamma_{j}\right)),g)\\ =\hat{e}(H(H\;I\;D||E\left(\gamma_{j}\right){)}^{S\;S\;K||T\;S},g)\\ =\hat{e}(H(H\;I\;D||E\left(\gamma_{j}\right)),g^{S\;S\;K||T\;S})\\ =\hat{e}(H(H\;I\;D||E\left(\gamma_{j}\right)),H\;I\;D) \end{array}.$$

If the certificate is valid, ${\mathrm{PC}}_{i}$ derives $E(\gamma_{j})$ from the message and decrypts the message illustrated as:

(12) $$\begin{array}{c} \;\;\;D(E\left(\gamma_{j}\right))\\ =S\;\_D\;E\;C_{hsk}(S\;\_E\;N\;C_{hsk}\left(T\;S|\right|\gamma_{j}))\\ =T\;S||\gamma_{j} \end{array},$$

where $S\;\_D\;E\;C_{hsk}\left(M\right)$ denotes symmetric decryption using hsk. At this point, the keying message $\gamma_{j}$ is securely transmitted.

●  **GrKeCom**: This algorithm is designed for group key derivation from the received keying message $\gamma_{j}$. In **GrKeCom**, a modulo division on the ${\mathrm{PC}}_{i}$ side is conducted as:

(13) $$P\;G\;K_{j}\equiv \gamma_{j}\;\mathrm{mod}\;P\;S\;K_{i},$$

where $P\;S\;K_{i}$ is the allocated secret key. As defined above,

$$\left\{\begin{array}{c} P\;G\;K_{j}<q<P\;S\;K_{i}<p\\ \mu \;\mathrm{mod}\;P\;S\;K_{i}\equiv 1 \end{array}\right.$$

holds, which guarantees that the derived group key $P\;G\;K_{j}$ is equal to the original one. At this point, the group key generation is finished. All the ${\mathrm{PC}}_{i}$ of department *j* share $P\;G\;K_{j}$ with HC.

### 4.3. PC Join and Leave Operations

In the practical scenario, patients frequently join or leave the department [4,45]. Assume patient $P_{i}$ of department *j* is restored to health after receiving the treatment. ${\mathrm{PC}}_{i}$ is not allowed to obtain the broadcast message after revocation for the purpose of privacy protection towards the remaining patients. Moreover, the newly joined patient needs to be allocated the group key. Consequently, the group key should always be updated when join or leave operations happen.

In this section, the key updating scheme is illustrated respectively from two aspects, namely the PC join operation and the PC leave operation. Note that we demonstrate the join and leave operations in the single-PC case. That is, only one PC is to join or leave the department at the same time. Subsequently, the scenario of multiple PCs joining and leaving the same department is studied in the batch updating operation phase. The detailed description of the join and leave operations, as well as the batch updating operation is as follows.

#### 4.3.1. PC Join Operation Phase

As mentioned above, the PC join operation in department *j* is considered in this section. It is obvious that the HC should update the group key $P\;G\;K_{j}$ as soon as a specific patient, named $P_{join}$, joins department *j*. We would like to emphasize that $P_{join}$ should register to HC first, which is in accordance with the actual situations. Then, $P_{join}$ is assigned ${\mathrm{PC}}_{join}$ and obtains its own necessary secret key set $\langle P\;S\;K_{join},H\;I\;D_{join},hsk\rangle$ from HC. Subsequently, **JoKeUpdate** is conducted by HC to generate the rekeying message of ${\mathrm{PC}}_{join}$ and other *n* PCs of department *j*. Finally, by conducting **JoKeDerive**, the updated group key is distributed to all the n+1 PCs of department *j*. The design of **JoKeUpdate** and **JoKeDerive** is described in detail below.

●  **JoKeUpdate**: The HC conducts **JoKeUpdate** to generate the rekeying message for both ${\mathrm{PC}}_{join}$ and the current *n* PCs. A few steps are necessary as introduced below: First, for ${\mathrm{PC}}_{join}$, HC computes its corresponding $x_{join}$ and $y_{join}$ according to the **PreCom** algorithm in Section 4.2. Hence, the variable $var_{join}$ can be computed as:

(14) $$var_{join}=x_{join}\times y_{join}.$$

In this way, HC computes the intermediate value $\mu_{join}$ defined as:

(15) $$\mu_{join}=\mu +var_{join}.$$

Consequently, HC selects a new group key $P\;G\;K_{j-join}$ and generates the rekeying message $\gamma_{j-join}$ by computing:

(16) $$\gamma_{j-join}=P\;G\;K_{j-join}\times \mu_{join}.$$

Thereafter, by conducting the **SecHtoP** algorithm introduced in Section 4.2, the rekeying message $\gamma_{j-join}$ can be securely transmitted to the n+1 PCs, which includes one new joining ${\mathrm{PC}}_{join}$ and existing *n* PCs of department *j*.

●  **JoKeDerive**: This algorithm is designed for the aforementioned n+1 PCs to derive the updated group key $P\;G\;K_{j-join}$ from $\gamma_{j-join}$. After the verification process through **AuthMess** in Section 4.2, the ${\mathrm{PC}}_{i\in [1,n]\cup \{join\}}$ conducts a modulo division, illustrated as:

(17) $$P\;G\;K_{j-join}\equiv \gamma_{j-join}\;\mathrm{mod}\;P\;S\;K_{i}.$$

Note that the secret key $P\;S\;K_{join}$ of ${\mathrm{PC}}_{join}$ is included in $\mu_{join}$ so that the derived new group key $P\;G\;K_{j-join}$ is equal to the original one. The process of **JoKeDerive** is similar to the group key derivation phase presented in Section 4.2.

#### 4.3.2. PC Leave Operation Phase

In this section, we assume that the patient $P_{leave}$ is restored to health. Hence, HC deletes this patient and the corresponding ${\mathrm{PC}}_{leave}$ from department *j*. Moreover, if some PCs in department *j* were compromised, HC would delete the compromised PC in the same way. In this case, the effective compromised detection strategy is necessary. As for this paper, some existing schemes can be applied in order to detect the compromised PCs periodically [46,47].

In this phase, HC conducts the **LeKeUpdate** algorithm first to generate the rekeying message $\mu_{j-leave}$ and transmits it to the remaining n−1${\mathrm{PC}}_{i\in [1,n]\backslash \{leave\}}$ securely. Then, **LeKeDerive** is adapted on the ${\mathrm{PC}}_{i}$ side. Hence, the updated group key $P\;G\;K_{j-leave}$ is derived by HC and the rest of the n−1 PCs. The design of **LeKeUpdate** and **LeKeDerive** is described in detail below.

●  **LeKeUpdate**: The HC conducts **LeKeUpdate** to generate the rekeying message concerning the remaining n−1 PCs. A few steps are necessary as introduced below: First, HC obtains $\mu_{leave}$ of ${\mathrm{PC}}_{leave}$ demonstrated as:

(18) $$\mu_{leave}=\mu -var_{leave},$$

where $var_{leave}$ is stored in HC’s memory. Consequently, HC selects a new group key $P\;G\;K_{j-leave}$ and computes the rekeying message $\gamma_{j-leave}$ according to:

(19) $$\gamma_{j-leave}=P\;G\;K_{j-leave}\times \mu_{leave}.$$

Thereafter, by conducting the **SecHtoP** algorithm introduced in Section 4.2, the rekeying message $\gamma_{j-leave}$ can be securely transmitted.

●  **LeKeDerive**: After the verification process with the **AuthMess** algorithm in Section 4.2, ${\mathrm{PC}}_{i\in [1,n]\backslash \{leave\}}$ conducts **LeKeDerive** to derive the updated group key $P\;G\;K_{j-leave}$, illustrated as:

(20) $$P\;G\;K_{j-leave}\equiv \gamma_{j-leave}\;\mathrm{mod}\;{P\;S\;K}_{i}.$$

Note that the secret key $P\;S\;K_{leave}$ of ${\mathrm{PC}}_{leave}$ is excluded in $\mu_{leave}$ so that the removed patient $P_{leave}$ cannot derive the correct group key. The process of **LeKeDerive** is similar to the group key derivation phase presented in Section 4.2.

#### 4.3.3. Batch Updating Phase

With the particular feature of CRT, batch updating for multiple PCs can be achieved accordingly, which meets the practical requirements for medical WBAN. In this section, we present the batch updating involving the join and leave operations of multiple PCs at the same time. Suppose that $P_{bj\in [1,w]}$ delegate *w* joining patients in department *j*. Similarly, $P_{bl\in [1,z]}$ denote *z* leaving patients at the same time. $P_{bj}$ and $P_{bl}$ are respectively combined with ${PC}_{bj}$ and ${\mathrm{PC}}_{bl}$. Hence, after updating, the number of PCs in department *j* is n+w−z.

In our design, first, HC conducts the **BaKeUpdate** algorithm to generate the batch rekeying message $\gamma_{j-batch}$ and uses **SecHtoP** to distribute it to all the n+w PCs. Afterwards, **AuthMess** is conducted for verification on the PC side. Finally, **BaKeDerive** is conducted so that the updated group key $P\;G\;K_{j-batch}$ is obtained by n+w−z PCs in department *j*. It is noteworthy that the **SecHtoP** and **AuthMess** algorithms are the same as the ones presented in Section 4.2. The design of **BaKeUpdate** and **BaKeDerive** is described in detail below.

●  **BaKeUpdate**: The HC conducts **BaKeUpdate** to generate the batch rekeying message for the n+w−z PCs. A few steps are necessary as introduced below: First, with the aforementioned **PreCom** algorithm described in Section 4.2, HC computes the corresponding $x_{bj}$ and $y_{bj}$ of *w*${\mathrm{PC}}_{bj\in [1,w]}$. Hence, the variable for ${\mathrm{PC}}_{bj}$ is obtained as:

(21) $$var_{bj}=x_{bj}\times y_{bj}.$$

Consequently, the sum $var_{b}^{+}$ involving all the *w* joining PCs can be computed as:

(22) $$\begin{array}{cc} var_{b}^{+} & =\sum_{bj=1}^{w}var_{bj}\\ & =\sum_{bj=1}^{w}\left(x_{bj}\times y_{bj}\right) \end{array}.$$

Similarly, the sum $var_{b}^{-}$ involving all the *z* leaving PCs can be computed as:

(23) $$\begin{array}{cc} var_{b}^{-} & =\sum_{bl=1}^{z}var_{bl}\\ & =\sum_{bl=1}^{z}\left(x_{bl}\times y_{bl}\right) \end{array}.$$

Hence, the intermediate value including *w* joining PCs and *z* leaving PCs is defined as follows:

(24) $$\mu_{j-ba}=\mu +var_{b}^{+}-var_{b}^{-}.$$

As a result, HC chooses a new group key $P\;G\;K_{j-batch}$ and generates the batch rekeying message $\gamma_{j-batch}$, demonstrated as:

(25) $$\gamma_{j-batch}=P\;G\;K_{j-batch}\times \mu_{j-ba}.$$

Afterwards, by conducting the **SecHtoP** algorithm introduced in Section 4.2, the batch rekeying message $\gamma_{j-batch}$ can be distributed to all the n+w PCs.

●  **BaKeDerive**: After the verification process using the **AuthMess** algorithm in Section 4.2, ${\mathrm{PC}}_{i\in [1,n+w]}$ derives the updated group key $P\;G\;K_{j-batch}$ from $\gamma_{j-batch}$ using **BaKeDerive**. The ${\mathrm{PC}}_{i\in [1,n+w-z]}$ conducts a modulo division, illustrated as:

(26) $$P\;G\;K_{j-batch}\equiv \gamma_{j-batch}\;\mathrm{mod}\;P\;S\;K_{i}.$$

Note that the *w* secret keys $P\;S\;K_{bj}$ of new join ${\mathrm{PC}}_{bj}$ are included in $\mu_{j-ba}$ so that the derived $P\;G\;K_{j-join}$ is equal to the original one. Additionally, the secret keys of ${\mathrm{PC}}_{bl\in [1,z]}$ are excluded in $\mu_{j-ba}$ so that the removed patient $P_{bl\in [1,z]}$ cannot get the correct group key.

At this point, the batch updating procedure interrelated with *w* joining patients and *z* leaving patients is completed. The group key for all the n+w−z PCs in department *j* is updated securely.

### 4.4. Group Key Generation for PC and Sensors

In this section, our design is motivated by coded cooperative data exchange (CCDE). Assume that *k* packages are loaded to *t* clients previously. In simple terms, the goal of CCDE is to recover the *k* packages for *t* clients in minimal transmission. Upon completion, each client obtains all the *k* packages. So far, many research achievements have been made on solving the CCDE problem. According to [38] and [48], if the *t* clients are fully connected, the CCDE problem can be solved in polynomial time. Inspired by the group key agreement designed in [5], we consider assigning in total *k* master keys to all the sensors in department *j*. The master key distribution follows the rule that every two sensors share at least one master key. Hence, the sensors of department *j* are fully connected with each other. With the assistance of the corresponding PC, the sensors can build the group key cooperatively. Based on Definition 1 in [5], the CCDE-based scheme is feasible for efficient sensor association for the purpose of achieving optimal transmission passes.

For a better description, we take a patient $P_{i}$ with ${\mathrm{PC}}_{i}$, for instance, where $P_{i}$ is in department *j*. Let $C_{i}=\left\{{\mathrm{SN}}_{v}|v\in \left[1,m\right],m\in N^{*}\right\}$ be a set of *m* wireless sensors allocated to $P_{i}$. The association of these *m* sensors will be conducted after ${\mathrm{PC}}_{i}$ successful registers to HC. The proposed sensor association scheme can be divided into two phases: the setup phase and the key generation phase. The setup phase is responsible for secret key allocation and some necessary preparation. Thereafter, the group key is generated in the next key generation phase. The detailed descriptions of these two phases are presented as follows.

#### 4.4.1. Setup Phase

In this phase, ${\mathrm{PC}}_{i}$ assigns necessary secret information to the *m* sensors. First, the ${\mathrm{PC}}_{i}$ conducts **SecKeDis** to generate temporary identity $P\;I\;D_{i}$ and symmetric secret key nsk. Thereafter, ${\mathrm{PC}}_{i}$ conducts **MasKeDis** to distribute the predefined master keys to sensor ${\mathrm{SN}}_{v\in [1,m]}$. The design of **SecKeDis** and **MasKeDis** is described in detail below.

●  **SecKeDis**: The ${\mathrm{PC}}_{i}$ conducts **SecKeDis** to generate nsk and $P\;I\;D_{i}$. Let $Z_{h}^{*}$ be a nonnegative integer set less than *h*, where *h* is assumed to be a large prime number. Additionally, $G_{T}$ is defined as a multiplicative group of *h*, and *u* is the generator of $G_{T}$. First, ${\mathrm{PC}}_{i}$ randomly chooses nsk from $Z_{h}^{*}$. Hence, $P\;I\;D_{i}$ is generated, illustrated as follows:

(27) $$P\;I\;D_{i}=u^{P\;S\;K_{i}\left|\right|T\;S},$$

where $P\;S\;K_{i}$ is the confidential information of ${\mathrm{PC}}_{i}$. Thereafter, ${\mathrm{PC}}_{i}$ stores $\langle P\;S\;K_{i},P\;I\;D_{i},nsk\rangle$ in its memory.

●  **MasKeDis**: The ${\mathrm{PC}}_{i}$ conducts **MasKeDis** to distribute a set of master keys among the *m* sensors. Let $Q_{i}=\left\{k_{h}|h\in \left[1,c\right],c>m\wedge c\in N^{*}\right\}$ be the *c* master keys to be allocated. According to our design, a master key subset $B_{v}$ denoted by $B_{v}\subseteq Q_{i}$ is distributed to ${\mathrm{SN}}_{v}\in C_{i}$. Hence, $\forall v_{1},v_{2}\in \left\{1,\ldots ,m\right\}$ ($v_{1}\neq v_{2}$), $B_{v_{1}}\cap B_{v_{2}}\neq ⌀$ and $B_{v_{1}}\cup B_{v_{2}}\subseteq Q_{i}$ hold. In this way, each sensor ${\mathrm{SN}}_{v}\in C_{i}$ shares at least one master key with each remaining sensor. Upon completion, ${\mathrm{PC}}_{i}$ assigns $\langle P\;I\;D_{i},nsk,B_{v}\rangle$ to sensor ${\mathrm{SN}}_{v}$.

#### 4.4.2. Key Generation Phase

In this phase, ${\mathrm{PC}}_{i}$ is responsible for distributing the keying message to all the sensors securely. First, ${\mathrm{PC}}_{i}$ conducts **${\mathrm{MasKeSel}}_{1}$** to select the most widely-shared master key $k_{\Psi }^{1}\in Q_{i}$ in all the *m* subsets $B_{v\in [1,m]}$ and computes the session key $S\;k_{\Psi }^{1}$. Afterwards, ${\mathrm{PC}}_{i}$ transmits the session key $S\;k_{\Psi }^{1}$ to sensors with **SecPtoS**. Subsequently, **AuthSess** is conducted by sensor ${\mathrm{SN}}_{v}\in C_{i}$ so as to guarantee the validity of the received session key and to compare it with $B_{v}$. Hence, the sensors preloaded with $k_{\Psi }^{1}$ are classified as one subset $\Lambda_{1}\subseteq C_{i}$. Other sensors without $k_{\Psi }^{1}$ abandon the received message.

Thereafter, ${\mathrm{PC}}_{i}$ runs **${\mathrm{MasKeSel}}_{2}$** to select the second master key $k_{\Psi }^{2}$. Similarly, the sensors preloaded with $k_{\Psi }^{2}$ are classified as the second subset $\Lambda_{2}\subseteq C_{i}$. According to our design, $\Lambda_{1}\cap \Lambda_{2}\neq ⌀$. In other words, at least one sensor is preloaded with both $k_{\Psi }^{1}$ and $k_{\Psi }^{2}$. Let ${\mathrm{SN}}_{\hbar }^{\Lambda_{1}\cap \Lambda_{2}}$ be the sensors such that ${\mathrm{SN}}_{\hbar }^{\Lambda_{1}\cap \Lambda_{2}}\in \Lambda_{1}\cap \Lambda_{2}$($\hbar \in [1,\Phi_{1}]$), assuming that there are in total $\Phi_{1}$ elements in $\Lambda_{1}\cap \Lambda_{2}$. Subsequently, ${\mathrm{SN}}_{\hbar \in [1,\Phi_{1}]}^{\Lambda_{1}\cap \Lambda_{2}}$ with both $k_{\Psi }^{1}$ and $k_{\Psi }^{2}$ conducts **GrKeEnc** so that the sensors in ${∁}_{\Lambda_{2}}\left(\Lambda_{1}\cap \Lambda_{2}\right)$ can derive the session key $S\;k_{\Psi }^{1}$. Note that $S\;k_{\Psi }^{1}$ is considered as the group key $S\;G\;K_{i}$.

Now, $S\;k_{\Psi }^{1}$ is distributed to the sensors in $\Lambda_{1}\cap \Lambda_{2}$. Subsequently, ${\mathrm{PC}}_{i}$ repeatedly conducts the above process in order to distribute $S\;k_{\Psi }^{1}$ to the remaining sensors in ${∁}_{C_{i}}\left(\Lambda_{1}\cup \Lambda_{2}\right)$. In this way, after several broadcast transmission passes, all the ${\mathrm{SN}}_{v}\in C_{i}$ can finally get $S\;k_{\Psi }^{1}$ as the group key. Hence, the key generation phase is completed. The design of **${\mathrm{MasKeSel}}_{1}$**, **SecPtoS**, **AuthSess**, **${\mathrm{MasKeSel}}_{2}$** and **GrKeEnc** is respectively described in detail below.

●  **${\mathrm{MasKeSel}}_{1}$**: This algorithm is designed for ${\mathrm{PC}}_{i}$ to select the master key $k_{\Psi }^{1}$. It is assumed that ${\mathrm{PC}}_{i}$ primarily chooses the master key involving more sensors. As a result, the corresponding session key $S\;k_{\Psi }^{1}$ is generated, illustrated as:

(28) $$S\;k_{\Psi }^{1}=H(k_{\Psi }^{1}||T\;S).$$

●  **SecPtoS**: After the computation of session key $S\;k_{\Psi }^{1}$, ${\mathrm{PC}}_{i}$ conducts **SecPtoS** for session key distribution. First, $S\;k_{\Psi }^{1}$ is encrypted by ${\mathrm{PC}}_{i}$ following:

(29) $$E_{1}\left(S\;k_{\Psi }^{1}\right)=S\;\_E\;N\;C_{nsk}(T\;S||S\;k_{\Psi }^{1}).$$

As illustrated before, $S\;\_E\;N\;C_{x}\left(M\right)$ denotes the symmetric encryption. Next, ${\mathrm{PC}}_{i}$ computes the certificate $S\;I\;G_{{P\;S\;K}_{i}}(T\;S||P\;I\;D_{i}\left|\right|E_{1}\left(S\;k_{\Psi }^{1}\right)$ according to Equation (9). Hence, the certificate $S\;I\;G_{{P\;S\;K}_{i}}(T\;S||P\;I\;D_{i}\left|\right|E_{1}\left(S\;k_{\Psi }^{1}\right))$ can be obtained by computing:

(30) $$S\;I\;G_{{P\;S\;K}_{i}}(T\;S||P\;I\;D_{i}\left|\right|E_{1}\left(S\;k_{\Psi }^{1}\right))=H(P\;I\;D_{i}\left|\right|E_{1}\left(S\;k_{\Psi }^{1}\right){)}^{P\;S\;K_{i}\left|\right|T\;S}.$$

After the above calculation, the message:

$$\langle T\;S||P\;I\;D_{i}\left|\right|E_{1}\left(S\;k_{\Psi }^{1}\right)||S\;I\;G_{{P\;S\;K}_{i}}(T\;S||P\;I\;D_{i}\left|\right|E_{1}\left(S\;k_{\Psi }^{1}\right))\rangle$$

is finally broadcast to ${\mathrm{SN}}_{v}\in C_{i}$. It is noteworthy that the entire process of **SecPtoS** is similar to the aforementioned **SecHtoP**.

●  **AuthSess**: This algorithm is designed for sensors to verify the received certificate from ${\mathrm{PC}}_{i}$. The whole process is similar to the aforementioned **AuthMess** algorithm. ${\mathrm{PC}}_{i}$ checks whether:

$$\begin{array}{c} \hat{e}(S\;I\;G_{{P\;S\;K}_{i}}(T\;S||P\;I\;D_{i}\left|\right|E_{1}\left(S\;k_{\Psi }^{1}\right)),u)\\ \overset{?}{=}\hat{e}(H(P\;I\;D_{i}\left|\right|E_{1}\left(S\;k_{\Psi }^{1}\right)),P\;I\;D_{i}) \end{array}$$

holds. The correctness is elaborated as follows:

(31) $$\begin{array}{c} \;\;\;\hat{e}(S\;I\;G_{{P\;S\;K}_{i}}(T\;S||P\;I\;D_{i}\left|\right|E_{1}\left(S\;k_{\Psi }^{1}\right)),u)\\ =\hat{e}(H({P\;I\;D}_{i}\left|\right|E_{1}\left(S\;k_{\Psi }^{1}\right){)}^{{P\;S\;K}_{i}\left|\right|T\;S},u)\\ =\hat{e}(H({P\;I\;D}_{i}\left|\right|E_{1}\left(S\;k_{\Psi }^{1}\right)),u^{P\;S\;K_{i}\left|\right|T\;S})\\ =\hat{e}(H({P\;I\;D}_{i}\left|\right|E_{1}\left(S\;k_{\Psi }^{1}\right)),P\;I\;D_{i}) \end{array}.$$

If the certificate is valid, ${\mathrm{SN}}_{v}$ derives $E_{1}\left(S\;k_{\Psi }^{1}\right)$ from the message and decrypts the message as:

(32) $$\begin{array}{c} \;\;\;D_{1}\left(E_{1}\left(S\;k_{\Psi }^{1}\right)\right)\\ =S\;\_D\;E\;C_{nsk}(S\;\_E\;N\;C_{nsk}(T\;S||S\;k_{\Psi }^{1})\\ =T\;S||S\;k_{\Psi }^{1} \end{array},$$

where $S\;\_D\;E\;C_{nsk}\left(M\right)$ denotes symmetric decryption using nsk. As a result, the keying message $S\;k_{\Psi }^{1}$ is securely transmitted.

●  **${\mathrm{MasKeSel}}_{2}$**: This algorithm is designed for ${\mathrm{PC}}_{i}$ to select the second master key $k_{\Psi }^{2}$. It is required that at least one sensor in $\Lambda_{1}$ stores master key $k_{\Psi }^{2}$ in its master key subset. That is, $\exists {\mathrm{SN}}_{\Omega }\in \Lambda_{1}$, $k_{\Psi }^{2}\in B_{\Omega }$ holds. Following this rule, ${\mathrm{PC}}_{i}$ chooses the master key involving more sensors in ${∁}_{C_{i}}\Lambda_{1}$. After that, session key $S\;k_{\Psi }^{2}$ is generated according to:

(33) $$S\;k_{\Psi }^{2}=H(k_{\Psi }^{2}||T\;S).$$

●  **GrKeEnc**: After ${\mathrm{PC}}_{i}$ broadcasts the session keys two times, sensors ${\mathrm{SN}}_{\hbar }^{\Lambda_{1}\cap \Lambda_{2}}$ have both $S\;k_{\Psi }^{1}$ and $S\;k_{\Psi }^{2}$. Consequently, ${\mathrm{SN}}_{\hbar \in [1,\Phi_{1}]}^{\Lambda_{1}\cap \Lambda_{2}}$ encrypts $S\;k_{\Psi }^{1}$ using $S\;k_{\Psi }^{2}$ as follows:

(34) $$E_{2}\left(S\;k_{\Psi }^{1}\right)=S\;\_E\;N\;C_{S\;k_{\Psi }^{2}}(T\;S||S\;k_{\Psi }^{1}).$$

Next, $E_{2}\left(S\;k_{\Psi }^{1}\right)$ is broadcast by ${\mathrm{SN}}_{\hbar \in [1,\Phi_{1}]}^{\Lambda_{1}\cap \Lambda_{2}}$. It is noteworthy that the transmission process is similar to the aforementioned **SecPtoS**. At last,

$$\langle T\;S||P\;I\;D_{i}\left|\right|E_{2}\left(S\;k_{\Psi }^{1}\right)||S\;I\;G_{{P\;S\;K}_{i}}(T\;S||P\;I\;D_{i}\left|\right|E_{2}\left(S\;k_{\Psi }^{1}\right))\rangle$$

is distributed.

After the message checking process employing **AuthSess**, sensors in ${∁}_{\Lambda_{2}}\left(\Lambda_{1}\cap \Lambda_{2}\right)$ derive the session key $S\;k_{\Psi }^{1}$. Hence, $S\;k_{\Psi }^{1}$ is distributed as the group key $S\;G\;K_{i}$.

The above process repeats until:

(35) $$\overset{\rho }{\underset{†=1}{⋃}}\Lambda_{†}=C_{i}$$

holds, where ρ denotes the transmission times on the ${\mathrm{PC}}_{i}$ side. At this point, the group key generation for PC and sensors is completed.

### 4.5. Sensor Join and Leave Operations

In this section, the occasions of sensor joining and leaving $C_{i}$ are considered respectively.

#### 4.5.1. Sensor Join Operation

In our system model, the sensor join operation should be available in order to offer continuous treatment for the current patient. Assume patient $P_{i}$ is equipped with *m* wireless sensors in department *j*. ${\mathrm{SN}}_{join}$ denotes the new sensor to be assigned. It is worth emphasizing that the existing *m* sensors have already been associated with ${\mathrm{PC}}_{i}$ through the generated group key $S\;G\;K_{i}$. In this case, the joining sensor ${\mathrm{SN}}_{join}$ first registers to ${\mathrm{PC}}_{i}$ and obtains $\langle P\;I\;D_{i},nsk,B_{join}\rangle$. Additionally, $B_{join}$ denotes the master key subset allocated to ${\mathrm{SN}}_{join}$. For $\forall \bar{v}\in \left[1,m\right]$, $B_{\bar{v}}\cap \;B_{join}\neq ⌀$ and $B_{\bar{v}}\cup \;B_{join}\subseteq Q_{i}$ hold. After that, ${\mathrm{PC}}_{i}$ selects the master key $k_{\Psi }^{join}$. Note that $k_{\Psi }^{join}$ is preloaded to $B_{join}$ and at least one existing sensor in $C_{i}$ simultaneously. That is, for $k_{\Psi }^{join}\in B_{join}$, $\exists \bar{v}\in \left[1,m\right]$, $k_{\Psi }^{join}\in B_{\bar{v}}\cap B_{join}$ holds. The next process for the joining sensor is similar to Section 4.4. As a result, all the m+1 sensors obtain the group key $S\;G\;K_{i}$. The sensor join operation is completed. Furthermore, the occasion with multiple sensors joining the group is similar to the above single-sensor case.

In conclusion, the above sensor join scheme emphasizes allocating the existing group key $S\;G\;K_{i}$ to the new join sensor. However, in order to enhance the security properties, the existing group key should be updated whenever a new sensor joins $C_{i}$, which is supported by the aforementioned group key generation process.

#### 4.5.2. Sensor Leave Operation

According to our system model, the sensors are assigned to each patient by the healthcare center and will not be frequently removed from the patient’s body. In most cases, the allocated sensors are combined with the related patient and keep working till the patient leaves the department. However, if the sensor is compromised or disabled, the current group key should be refreshed in timely manner. It is notable that in our design, the sensors are closely attached to or in the patient’s body so that the sensors are fully controlled by the patient, where the patient is assumed to be a benign user. Hence, for security consideration, ${\mathrm{PC}}_{i}$ should assign a new secret message and conduct the group key generation process again.

## 5. Security Analysis

In this section, we analyze the security properties of the proposed protocol. The security theorems, as well as the corresponding proofs are given below.

### 5.1. Resistance to Replay Attack

The adversary can conduct a replay attack by reusing the previous messages [49,50]. We analyze the resistance to replay attack in the proposed protocol.

**Theorem** **1.** *During the authentication process in the group key management between HC and PCs, replay attack can be prevented. That is, the reuse of the previous message sent from HC cannot pass the current authentication process on the PC side.*

**Proof** **of** **Theorem** **1.** The security of replay attack resistance is formally defined through game $G_{1}$. Let $A_{1}$ be a probabilistic time adversary. $C_{1}$ denotes the challenger, and *h* and *H* denote the random oracles. It is worth emphasizing that $C_{1}$ has the ability to simulate all the oracles and to output the signing message as a real signer [2,3]. In $G_{1}$, it is assumed that $A_{1}$ can conduct the following corresponding queries to $C_{1}$:

*h* query:$A_{1}$ can query the random oracle *h* at any time. $C_{1}$ simulates this random oracle by maintaining a list $L_{h}$ of tuple $\left\{j,{\mathrm{PC}}_{i}\right\}$, where $L_{h}$ is initialized to be empty. When the oracle is queried with input *j*, if the query *j* is already in $L_{h}$, $C_{1}$ outputs ${\mathrm{PC}}_{i}$ to $A_{1}$. Otherwise, $C_{1}$ generates a random ${\mathrm{PC}}_{i}$ and returns it to $A_{1}$. Note that $\left\{j,{\mathrm{PC}}_{i}\right\}$ is added to $L_{h}$.

Extract query: Upon receiving the query from $A_{1}$, $C_{1}$ executes the **SecKeGen** algorithm to generate relevant secret information $\left\{T\;S,P\;S\;K_{i},S\;S\;K,g,hsk\right\}$. It is notable that TS denotes the current time stamp. After that, $C_{1}$ computes HID and $E(\gamma_{j})$. Finally, $\left\{{\mathrm{PC}}_{i},H\;I\;D,T\;S,g,E\left(\gamma_{j}\right)\right\}$ is returned to $A_{1}$.

*H* query: $A_{1}$ can query the random oracle *H* at any time. $C_{1}$ simulates this random oracle *H* by maintaining a list $L_{H}$ of tuple $\left\{{\mathrm{PC}}_{i},Y_{i}\right\}$, where $L_{h}$ is initialized to be empty. When the oracle is queried with input ${\mathrm{PC}}_{i}$, if the ${\mathrm{PC}}_{i}$ is already in $L_{h}$, $C_{1}$ outputs ${\mathrm{PC}}_{i}$ to $A_{1}$. Otherwise, $C_{1}$ generates a random number $Y_{i}$ and returns it to $A_{1}$. Meanwhile, $\left\{{\mathrm{PC}}_{i},Y_{i}\right\}$ is added to $L_{h}$.

SigGen query: $C_{1}$ simulates the signature oracle by responding to the signature query of message $E(\gamma_{j})$. $C_{1}$ executes the **SecHtoP** algorithm to generate the signature $S\;I\;G(T\;S||H\;I\;D||E\left(\gamma_{j}\right))$ and return it to $A_{1}$.

Replay query: Upon receiving the signature from $A_{1}$, $C_{1}$ simulates the replay operation by conducting the **AuthMess** algorithm to check the validity of the received signature. The received signature is compared with the newly-generated signature after a certain time interval Δt by replaying the process.

As a result, $A_{1}$ obtains the signature $S\;I\;G(T\;S||H\;I\;D||E\left(\gamma_{j}\right))$, where the generated signature is valid and the following equation:

(36) $$\begin{array}{c} \;\;\;\hat{e}(S\;I\;G(T\;S||H\;I\;D||E\left(\gamma_{j}\right)),g)\\ =\hat{e}(H(H\;I\;D||E\left(\gamma_{j}\right){)}^{S\;S\;K||T\;S},g)\\ =\hat{e}(H(H\;I\;D||E\left(\gamma_{j}\right)),g^{S\;S\;K||T\;S})\\ =\hat{e}\left(Y_{i},H\;I\;D\right) \end{array}$$

holds. At this point, $\langle T\;S||H\;I\;D||E\left(\gamma_{j}\right)||S\;I\;G(T\;S||H\;I\;D||E\left(\gamma_{j}\right))\rangle$ is obtained by $A_{1}$, while the newly-generated signature $S\;I\;G(T\;S||g^{S\;S\;K||{T\;S}_{\Delta t}}||E\left(\gamma_{j}\right))$ involving the corresponding information satisfies:

(37) $$\begin{array}{c} \;\;\;\hat{e}\left(S\;I\;G(T\;S|\right|g^{S\;S\;K||{T\;S}_{\Delta t}}||E\left(\gamma_{j}\right)),g)\\ =\hat{e}(H(g^{S\;S\;K||T\;S_{\Delta t}}||E\left(\gamma_{j}\right){)}^{S\;S\;K||T\;S_{\Delta t}},g)\\ =\hat{e}\left({Y_{i}}^{\prime },H\;I\;D^{\prime }\right) \end{array},$$

where $T\;S_{\Delta t}$ is the time stamp at time Δt generated by $C_{1}$ ($T\;S_{\Delta t}>T\;S$). Accordingly, by conducting the replay query, $C_{1}$ runs the **AuthMess** algorithm as follows:

(38) $$\begin{array}{c} \;\;\;\hat{e}(S\;I\;G(T\;S_{\Delta t}||H\;I\;D||E\left(\gamma_{j}\right)),g)\\ =\hat{e}(H(g^{S\;S\;K||T\;S}||E\left(\gamma_{j}\right){)}^{S\;S\;K||T\;S_{\Delta t}},g)\\ =\hat{e}\left(Y_{i},H\;I\;D^{\prime }\right) \end{array}.$$

It is obvious that the reused previous signature can pass the authentication only when $Y_{i}=Y_{i}^{\prime }$ and $H\;I\;D=H\;I\;D^{\prime }$. That is, $T\;S_{\Delta t}=T\;S$, which contradicts the aforementioned definition. Hence, the replay attack is not available in the proposed group key management scheme between HC and PCs. □

**Theorem** **2.** *During the authentication process in the group key management between PC and sensors, the replay attack can be prevented. That is, the reuse of the previous message sent from PC cannot pass the current authentication process on the sensor side.*

**Proof** **of** **Theorem** **2.** The proof of Theorem 2 is similar to the above proof of Theorem 1. □

### 5.2. Resistance to Forgery Attack

In this section, we analyze the resistance to the forgery attack of the proposed protocol.

**Theorem** **3.** *The proposed group key management scheme between HC and PCs is existentially unforgeable in the random oracle model.*

**Proof** **of** **Theorem** **3.** Similarly, the proof of forgery attack resistance is formally defined through game $G_{2}$. Let $A_{2}$ be a probabilistic time adversary. $C_{2}$ denotes the challenger, and *h* and *H* denote the random oracles. It is worth noting that $C_{2}$ has the ability to simulate all the oracles and to output the signing message as a real signer. In $G_{2}$, it is assumed that $A_{2}$ can conduct the following corresponding queries to $C_{2}$:

*h* query: This is the same as the definition in Theorem 1.

Extract query: Upon receiving the query from $A_{2}$, $C_{2}$ executes the **SecKeGen** algorithm to generate relevant secret information $\left\{T\;S,P\;S\;K_{i},S\;S\;K,g,hsk\right\}$. Note that TS denotes the current time stamp. $\left\{{\mathrm{PC}}_{i},H\;I\;D,T\;S,g\right\}$ is returned to $A_{2}$.

SyEnc query: $C_{2}$ maintains a list $L_{S}$ of tuple $\left\{{\mathrm{PC}}_{i},\gamma_{j},E\left(\gamma_{j}\right)\right\}$, where $L_{S}$ is initialized to be empty. When queried by $A_{2}$, $C_{2}$ generates a random number as $\gamma_{j}$ and checks the list $L_{S}$. If $\left\{{\mathrm{PC}}_{i},\gamma_{j}\right\}$ is already in $L_{S}$, $C_{2}$ randomly chooses another value again. Otherwise, $C_{2}$ computes $E(\gamma_{j})$ with hsk. Finally, $\left\{{\mathrm{PC}}_{i},\gamma_{j},E\left(\gamma_{j}\right)\right\}$ is returned to $A_{2}$ and also added to $L_{S}$.

*H* query: This is the same as the definition in Theorem 1.

SigGen query: This is the same as the definition in Theorem 1.

Replay query: Upon receiving the signature from $A_{2}$, $C_{2}$ simulates the replay operation by conducting the **AuthMess** algorithm to check the validity of the received signature. The received signature is compared with the newly-generated signature of $E(\gamma_{j}^{\prime })$ ($\gamma_{j}^{\prime }\neq \gamma_{j}$).

Finally, the adversary $A_{2}$ obtains the signature $S\;I\;G(T\;S||H\;I\;D||E\left(\gamma_{j}\right))$, as well as $\left\{T\;S,H\;I\;D,E\left(\gamma_{j}\right)\right\}$ of ${\mathrm{PC}}_{i}$ by querying $C_{2}$. As a result, the equation $\hat{e}(S\;I\;G(T\;S||H\;I\;D||E\left(\gamma_{j}\right)),g)=\hat{e}\left(Y_{i},H\;I\;D\right)$ holds. Furthermore, $C_{2}$ outputs another signature $S\;I\;G(T\;S||H\;I\;D||E\left(\gamma_{j}^{\prime }\right))$ to $A_{2}$. Assume the signature can pass the authentication, illustrated as:

(39) $$\begin{array}{c} \;\;\;\hat{e}(S\;I\;G(T\;S||H\;I\;D||E\left(\gamma_{j}^{\prime }\right)),g)\\ =\hat{e}(H(H\;I\;D||E\left(\gamma_{j}^{\prime }\right){)}^{S\;S\;K^{\prime }\left|\right|T\;S},g)\\ =\hat{e}(H(H\;I\;D||E\left(\gamma_{j}^{\prime }\right)),g^{S\;S\;K^{\prime }\left|\right|T\;S})\\ =\hat{e}(H(H\;I\;D||E\left(\gamma_{j}^{\prime }\right)),H\;I\;D^{\prime }) \end{array}.$$

Thus, the forged signature can pass the authentication only when $Y_{i}=Y_{i}^{\prime }$ and $H\;I\;D=H\;I\;D^{\prime }$. That is, $g^{S\;S\;K^{\prime }\left|\right|T\;S}=H\;I\;D^{\prime }$, so that $S\;S\;K=S\;S\;K^{\prime }$, which contradicts the aforementioned assumption. Hence, the forgery according to the acquired message is not available in the proposed group key management scheme between HC and PCs. □

**Theorem** **4.** *The proposed group key management scheme between PC and sensors is existentially unforgeable in the random oracle model.*

**Proof** **of** **Theorem** **4.** The proof of Theorem 4 is similar to the above proof of Theorem 3. □

### 5.3. Forward Security

In this section, we analyze the forward security property of the proposed protocol.

**Theorem** **5.** *The proposed group key management scheme between HC and PCs provides forward security against an adversary. That is, the revoked PCs (patients) cannot get access to the current communication.*

**Proof** **of** **Theorem** **5.** This theorem is analyzed through game $G_{3}$. Let $A_{3}$ be the adversary by colluding with the revoked ${\mathrm{PC}}_{i}$ in department *j*. It is worth noting that $A_{3}$ obtains all the secret information stored in ${\mathrm{PC}}_{leave}$ and wants to derive the current group key $P\;G\;K_{j-leave}$. After receiving the keying message $\gamma_{j-leave}=P\;G\;K_{j-leave}\times \mu_{leave}$ from HC, $A_{3}$ conducts the modulo division to derive the group key. However, as described in the aforementioned sections, for the revoked ${\mathrm{PC}}_{leave}$, HC subtracts $var_{leave}$ from μ so that the $\mu_{leave}=\mu -var_{leave}$. In this case, the rekeying message only involves information of the rest of the n−1 PCs. Hence, the revoked ${\mathrm{PC}}_{leave}$ cannot derive the correct group key. That is, $P\;G\;K_{j-leave}\neq \gamma_{j-leave}\;\mathrm{mod}\;P\;S\;K_{leave}$. Thereafter, the rest of the n−1 PCs in department *j* can update their new group key securely. We assume that the size of $P\;S\;K_{leave}$ is ϖ bits. As a result, $A_{3}$ has to perform $2^{\varpi }$ times in order to obtain one $P\;S\;K_{i}$ of the rest of the n−1 PCs. Accordingly, the probability that $A_{3}$ can successfully obtain $P\;G\;K_{j-leave}$ is $\frac{n-1}{2^{\varpi }}$. Thus, the forward security is provided in our protocol between HC and PCs. □

**Theorem** **6.** *The proposed group key management scheme between PC and sensors provides forward security against an adversary. That is, the revoked sensors cannot get access to the current communication.*

**Proof** **of** **Theorem** **6.** As illustrated above, the sensors are closely attached on or in the patient’s body and are fully controlled by the patient. Assume a sensor is removed from the patient’s body. In this case, ${\mathrm{PC}}_{i}$ assigns new secret messages including $P\;I\;D_{i}$, nsk and a master key subset to the remaining sensor. The whole group key generation process will be conducted again to refresh the group key. In this way, the revoked sensor cannot derive the new group key since the vital secret information is different. □

### 5.4. Resistance to Collusion Attack

In this section, we analyze the collusion attack resistance of the proposed protocol.

**Theorem** **7.** *The proposed group key management scheme between PC and sensors provides forward security against an adversary. That is, the revoked sensors cannot get access to the current communication.*

**Proof** **of** **Theorem** **7.** We define the collusion attack through game $G_{4}$. Let $A_{4}^{1}$ and $A_{4}^{2}$ be the adversaries removed from department *j* at time $t_{1}$ and $t_{2}$ ($t_{1}<t_{2}$), respectively. At time $t_{1}$, $A_{4}^{1}$ leaves the department with the acquired group key $P\;G\;K_{t_{1}-}$. Meanwhile, the rekeying message $\gamma_{t_{1}}$ is obtained by $A_{4}^{2}$. Additionally, the updated group key $P\;G\;K_{t_{1}+}$ is derived by $A_{4}^{2}$. Similarly, at time $t_{2}$, $A_{4}^{2}$ leaves the department with the acquired group key $P\;G\;K_{t_{2}-}$ ($P\;G\;K_{t_{1}+}=P\;G\;K_{t_{2}-}$). The rekeying message $\gamma_{t_{2}}$ is obtained by $A_{4}^{2}$. Then, $A_{4}^{1}$ and $A_{4}^{2}$ exchange the obtained information to derive the updated group key $P\;G\;K_{t_{2}+}$. In this way, $A_{4}^{1}$ and $A_{4}^{2}$ are aware of $\langle P\;S\;K_{A_{4}^{1}},P\;S\;K_{A_{4}^{2}},P\;G\;K_{t_{1}-},P\;G\;K_{t_{1}+},\gamma_{t_{1}},\gamma_{t_{2}}\rangle$. With the above information, $P\;G\;K_{t_{2}+}$ is computed according to $P\;G\;K_{t_{2}+}=\gamma_{t_{2}}\;\mathrm{mod}\;P\;S\;K_{A_{4}^{\eta }}$ with η∈{1,2}. Assume the size of $P\;S\;K_{A_{4}^{\eta }}$ is ϖ bits. The probability that $A_{4}^{1}$ and $A_{4}^{2}$ can successfully obtain the group key is $\frac{n-2}{2^{\varpi }}$. Hence, the collusion attack is prevented. □

## 6. Performance Analysis

In this section, we present the performance analysis towards the proposed protocol. As illustrated in the above sections, our scheme consists of two parts: the group key management between HC and PCs and group key management between PC and sensors. The performances of the two schemes are respectively considered. Subsequently, the corresponding simulations and results are presented.

### 6.1. Group Key Management between HC and PCs

The proposed protocol is compared with two state-of-the-art grouping key management protocols: ESSA [4] and DAKM [44]. The comparison of the computational cost and storage, as well as the communication cost are demonstrated as follows.

#### 6.1.1. Computational Cost and Storage

The computational cost is defined as the total time consumption for group key generation [44]. Additionally, the storage mentioned here refers to the required memory size for the corresponding operations. The comparison result with ESSA and DAKM is given in Table 2. We denote the modulo operation as mod, the exponential operation as Ex and the bilinear pairing as *e*. Enc and Dec refer to encryption and decryption. Additionally, *H*, *M*, *D* and *A* represent the one-way hash function, multiplication operation, division operation and addition operation, respectively. Finally, the point multiplication operation is defined as *p*.

#### 6.1.2. Communication Cost

The communication cost refers to the time consumption for message transmission. The comparison result on communication cost is given in Table 3. Accordingly, both DAKM and our protocol require one broadcast for the whole process, which is efficient for resource-constrained wireless sensors.

### 6.2. Group Key Management between PC and Sensors

In this section, the proposed protocol is analyzed and compared with the ESSA protocol [4]. The comparisons of the computational cost and storage, as well as the communication cost are illustrated as follows.

#### 6.2.1. Computational Cost and Storage

The comparison result with ESSA [4] on the computational cost and storage is given in Table 4. The notations used in the table are the same as those in Table 2. As illustrated above, the sensors in subset $\Lambda_{M}\subseteq C_{i}$ get the session key $S\;k_{\Psi }^{M}$. Note that the process repeats for ρ times so that M∈[1,ρ]. For abetter description, we assume that there are $\Theta_{M}$ sensors in $\Lambda_{M}$. Meanwhile, there are $\Phi_{M}$ sensors in subset $\Lambda_{M}\cap \Lambda_{M+1}$. In this case, the computational cost on the ${\mathrm{PC}}_{i}$ side is (ρ+1)Ex+2ρH+ρEnc.

On the sensor side, we consider the average required computation for message authentication and encryption. The detailed procedures are as follows: First, after receiving the first message from ${\mathrm{PC}}_{i}$, the computation for each sensor in subset $\Lambda_{1}$ is 1e+1H+1Dec so that the total computation is $\Theta_{1}\left(1e+1H+1Dec\right)$. Similarly, in the second round, after receiving the message from ${\mathrm{PC}}_{i}$, the computation for all the $\Theta_{2}$ sensors in subset $\Lambda_{2}$ is $\Theta_{2}\left(1e+1H+1Dec\right)$. After that, $\Phi_{1}$ sensors in $\Lambda_{1}\cap \Lambda_{2}$ broadcast the message to others with computation 1Enc+1H+1Ex. Next, the $\Theta_{2}-\Phi_{1}$ sensors in ${∁}_{\Lambda_{2}}\left(\Lambda_{1}\cap \Lambda_{2}\right)$ computes for 1e+1H+1Dec. Hence, we can conclude that the total computation in the *i*-th rounds is:

(40) $$\begin{array}{c} \;\;\;\Theta_{i}\left(1e+1H+1Dec\right)+\Phi_{i-1}\left(1Enc+1H+1Ex\right)\\ \;\;\;+\left(\Theta_{i}-\Phi_{i-1}\right)\left(1e+1H+1Dec\right)\\ =\left(2\Theta_{i}-\Phi_{i-1}\right)e+2\Theta_{i}H+\left(2\Theta_{i}-\Phi_{i-1}\right)Dec\\ \;\;\;+\Phi_{i-1}Enc+\Phi_{i-1}Ex \end{array}.$$

In conclusion, the total computational cost for all the sensors is computed according to:

(41) $$\begin{array}{c} \;\;\;\mathrm{Comp}(i)\\ =\left(\Theta_{1}e+\Theta_{1}H+\Theta_{1}Dec\right)+\sum_{i=2}^{\rho }[\left(2\Theta_{i}-\Phi_{i-1}\right)e+2\Theta_{i}H\\ \;\;\;+\left(2\Theta_{i}-\Phi_{i-1}\right)Dec+\Phi_{i-1}Enc+\Phi_{i-1}Ex]\\ \approx \Theta_{1}\left(e+H+Dec\right)+2\left(e+H+Dec\right)\sum_{i=2}^{\rho }\Theta_{i}\\ =\left(e+H+Dec\right)\left(\Theta_{1}+2\sum_{i=2}^{\rho }\Theta_{i}\right) \end{array}.$$

Consequently, the average computational cost on the sensor side is:

(42) $$\begin{array}{c} \;\;\;\mathrm{AveComp}\_\mathrm{Sen}(i)\\ =\frac{\mathrm{Comp}(i)}{m}\\ =\left[\left(e+H+Dec\right)\left(\Theta_{1}+2\sum_{i=2}^{\rho }\Theta_{i}\right)\right]/m \end{array}.$$

We consider the extreme situation where ${\mathrm{PC}}_{i}$ needs to conduct m−1 broadcasting. In this assumption, the computational cost reaches the upper limitation. That is,

$$\left\{\begin{array}{c} \rho =m-1\\ \Theta_{i}=2,i\in \left\{1,\ldots ,\rho \right\} \end{array}.\right.$$

In this way, the maximum average computation cost on the sensor side is:

(43) $$\begin{array}{c} \;\;\;\mathrm{AveComp}\_\mathrm{Sen}(i)\\ =\frac{\mathrm{Comp}(i)}{m}\\ =(e+H+Dec)(2+4(m-2))/m\\ =\left(4-\frac{6}{m}\right)\left(e+H+Dec\right) \end{array}.$$

According to the practical requirement, m≫6; thus:

AveComp_Sen(i)≈4(e+H+Dec).

Subsequently, the storage comparison with ESSA is shown in Table 4. It is notable that the value $k_{P\;S\;K^{i}}$ in the table denotes a certain storage allocated for the preloaded master keys on the both PC and sensor side.

After this comparison with the existing two protocols on the group key management in WBAN, the simulations for the three protocols are presented, so as to prove the efficiency of the proposed protocol.

Subsequently, the storage comparison with ESSA is shown in Table 4. It is notable that the value *k* in the table denotes a certain storage allocated for the preloaded master keys on both the PC and sensor side. The comparison result shows that our protocol requires less memory size compared with the ESSA protocol.

#### 6.2.2. Communication Cost

The comparison result on the communication cost is given in Table 5. In ESSA [4], the transmission type during the authentication between PC and sensors is unicast. After that, broadcast is used for group key derivation. ${\mathrm{PC}}_{i}$ communicates with each sensor for four rounds. Hence, the total communication cost is 4m+1. As described above, ${\mathrm{PC}}_{i}$ broadcasts for ρ times to assign necessary messages to sensors. Moreover, each sensor in subset $\Lambda_{M}\cap \Lambda_{M+1}$ (M∈[1,ρ−1]) broadcasts the keying message to other sensors. In this way, the total communication cost is $\rho +\sum_{i=1}^{\rho -1}\Phi_{i}$. Similar to the above section, we set ρ=m−1 and $\Phi_{i}=2$, i∈{1,…,ρ−1} to compute the maximum communication cost. That is,

(44) $$\begin{array}{c} \;\;\;\rho +\sum_{i=1}^{\rho -1}\Phi_{i}\\ =m-1+2(m-2)\\ =3m-5 \end{array}.$$

In this case, we can get 3m−5<4m+1. It is obvious that our protocol requires less communication cost for group key management between PC and sensors.

### 6.3. Simulation Experiments and Results

In the previous two sections, adequate performance analysis and comparison emphasizing computational and communication cost are provided, along with a mathematical discussion and estimation for extreme cases. In addition, relevant simulations are presented in order to prove the efficiency of our protocol. It is worth noting that the time consumption for group key generation and distribution is particularly concerned, which is the crucial factor in the performance evaluation of WBANs.

The experiments were conducted on Windows 10 with a 2.70-GHz Intel(R) Core i7-6820HK CPU and 16 GB memory. Two parts of the proposed protocol, namely the group key management between HC and PCs and group key management between PC and sensors, were performed in Visual Studio 2015 with C++ language. Moreover, the Pairing-Based Cryptography (PBC) library was adopted accordingly.

**Figure 2:**
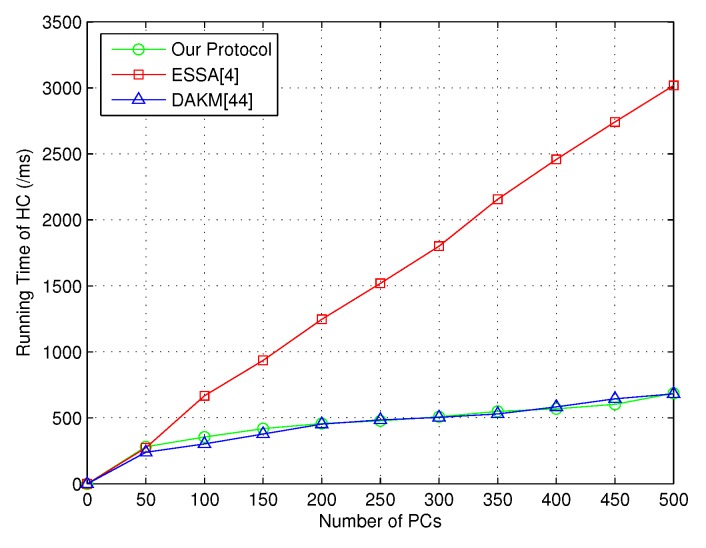
Time consumption of HC for key generation between HC and PCs.

**Figure 3:**
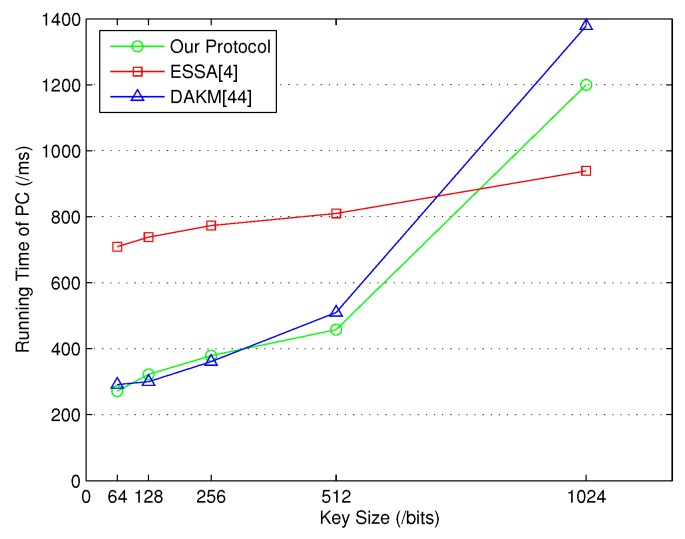
Time consumption of PC for key generation between HC and PCs.

The experiments on group key management between HC and PCs were conducted first. Note that the assignment of necessary secret information was designed to be done before the formal group key generation. Hence, the time consumption for **SecKeGen** was not included. The simulation was performed for several times based on different numbers of PCs. The comparison results with ESSA [4] and DAKM [44] are presented in Figure 2 and Figure 3. As shown in Figure 2, it is obvious that our protocol required less running time.

When the number of PCs increased, the running time for our protocol and DAKM [44] was similar. Additionally, the running time for each PC was affected by the key size, where in Figure 3, our protocol obviously required less running time on the PC side when the key size was set to 512 bits.

**Figure 4:**
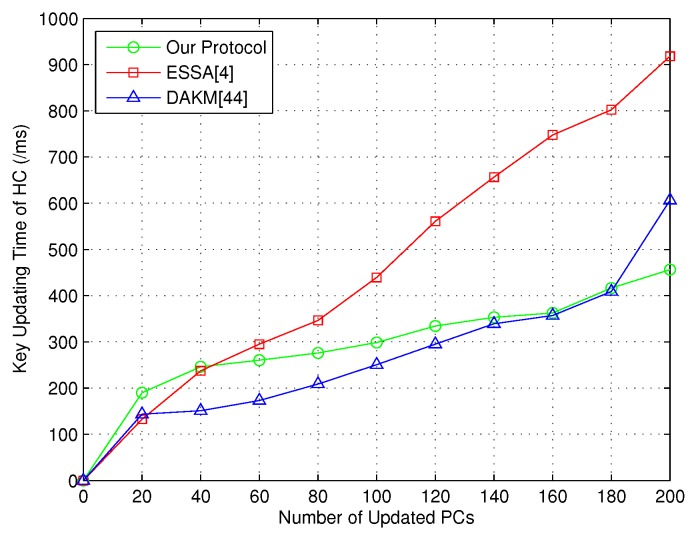
Key updating time of HC.

After that, the group key updating time of HC was considered in order to prove the efficiency of our group key updating scheme based on CRT. Note that both the joining and revoked PCs were defined to be the updated PCs. In this way, the key updating time is shown in Figure 4.

**Figure 5:**
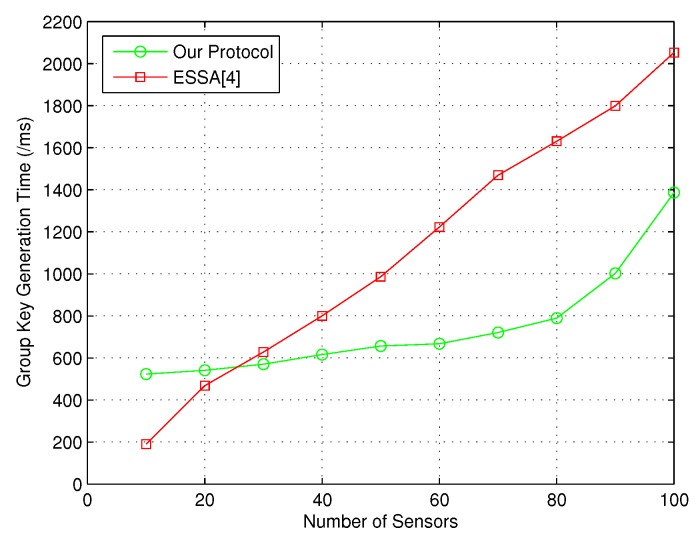
Group key generation time between PC and sensors.

Similarly, the comparison result with ESSA [4] on group key generation time between PC and sensors is given in Figure 5.

In a word, the above simulation results demonstrate that our protocol could provide better performance than the state-of-the-art group key management protocols.

## 7. Conclusions

In this paper, first, a novel practical WBAN system model with a notification channel is designed. Moreover, an efficient group key management protocol employing the Chinese remainder theorem (CRT) between HC and PCs is introduced, which supports secure group key updating. In this way, the HC is capable of broadcasting the message to different patient groups. Additionally, the group key scheme between PC and sensors is designed, which is motivated by coded cooperative data exchange (CCDE). Formal security analysis is given, indicating that the proposed protocol can achieve the desired security properties. Furthermore, performance analysis demonstrates that the proposed protocol is efficient compared with the state-of-the-art.

## Figures and Tables

**Table 1:** Notations.

Notation	Description
HC, PC	Healthcare center, personal controller
$P_{i}$	Patient
hsk, nsk	Symmetric secret key
$P\;S\;K_{i}$	Secret key of ${\mathrm{PC}}_{i}$
SSK	HC master key
HID, $P\;I\;D_{i}$	HC and ${\mathrm{PC}}_{i}$ temporary identity
*g*, *u*	Generators of G and $G_{T}$
TS	Time stamp
*n*	Number of patients in department *j*
$P\;G\;K_{j}$	Group key for HC and PCs in department *j*
$S\;\_E\;N\;C_{x}\left(M\right)$	Symmetric encryption on *M* with *x*
$S\;\_D\;E\;C_{x}\left(M\right)$	Symmetric decryption on *M* with *x*
$S\;I\;G_{x}\left(T\;S||M\right)$	Signature on *M*
*m*	Number of sensors attached to $P_{i}$
H()	One-way hash function
$B_{v}$	Master key subset preloaded to ${\mathrm{SN}}_{v}$
$k_{\Psi }^{i}$	Shared master key
$S\;k_{\Psi }^{i}$	Session key
$\Lambda_{i}$	Sensors preloaded with $k_{\Psi }^{i}$
$S\;G\;K_{i}$	Sensor group key of $P_{i}$
ρ	Transmission times on the ${\mathrm{PC}}_{i}$ side
$\Theta_{i}$	Number of sensors in $\Lambda_{i}$
$\Phi_{i}$	Number of sensors in $\Lambda_{i}\cap \Lambda_{i+1}$

**Table 2:** Comparison of computational cost and storage.

Protocol	ESSA [4]	DAKM [44]	Our Protocol
Computation of HC	np + nmod + 2nA + 2nM + nH	3Enc + 2nM + nD + (n−1)*A*	2Ex + 2nM + nD + 1Enc + 1H + (n−1)*A*
Computation of PC	1p + 1mod + 2A + 2M + 1H	1Dec + 1mod + 1Enc	1e + 1H + 1Dec + 1mod
Storage of HC	3n + 10	5n + 9	3n + 10
Storage of PC	13	10	8

**Table 3:** Comparison of the communication cost.

Protocol	ESSA [4]	DAKM [44]	Our Protocol
Transmission Type	Unicast	Broadcast	Broadcast
Communication Cost	3n	1	1

**Table 4:** Comparison of the computational cost and storage.

Protocol	ESSA [4]	Our Protocol
Computation of PC	(2m + 1)*p* + 6mH + (m−1)*A* + Enc	(ρ + 1)Ex + 2ρH + ρEnc
Computation of Sensor	2p + 6H + Dec	$\left[\left(e+H+Dec\right)\left(\Theta_{1}+2\sum_{i=2}^{\rho }\Theta_{i}\right)\right]/m$
Storage of PC	6m + 9	km + 8
Storage of Sensor	15	9 + *k*

**Table 5:** Comparison of communication cost.

Protocol	ESSA [4]	Our Protocol
Transmission Type	Unicast/Broadcast	Broadcast
Communication Cost	4m + 1	$\rho +\sum_{i=1}^{\rho -1}\Phi_{i}$
